# A Retrospective Case Series of Thiamine Deficiency in Non-Alcoholic Hospitalized Veterans: An Important Cause of Delirium and Falling?

**DOI:** 10.3390/jcm10071449

**Published:** 2021-04-01

**Authors:** Elisabeth Mates, Deepti Alluri, Tailer Artis, Mark S. Riddle

**Affiliations:** 1 Medicine Department, Veterans Affairs Sierra Nevada Healthcare System, Reno, NV 89502, USA; Mark.Riddle654@va.gov; 2 School of Medicine, University of Nevada, Reno, NV 89502, USA; tartis@med.unr.edu; 3 Sound Physicians, Lutheran Hospital, Fort Wayne, IN 46804, USA; dalluri89@gmail.com

**Keywords:** thiamine deficiency, delirium, falls, thiamine deficiency symptoms, encephalopathy, inpatient, vitamin B1, beriberi, gastrointestinal dysfunction

## Abstract

Thiamine deficiency (TD) in non-alcoholic hospitalized patients causes a variety of non-specific symptoms. Studies suggest it is not rare in acutely and chronically ill individuals in high income countries and is underdiagnosed. Our aim is to demonstrate data which help define the risk factors and constellation of symptoms of TD in this population. We describe 36 cases of TD in hospitalized non-alcoholic veterans over 5 years. Clinical and laboratory data were extracted by chart review +/− 4 weeks of plasma thiamine level 7 nmol/L or less. Ninety-seven percent had two or more chronic inflammatory conditions (CICs) and 83% had one or more acute inflammatory conditions (AICs). Of possible etiologies of TD 97% had two or more of: insufficient intake, inflammatory stress, or increased losses. Seventy-five percent experienced 5% or more weight loss. Ninety-two percent had symptoms with the most common being weakness or falling (75%) followed by neuropsychiatric manifestations (72%), gastrointestinal dysfunction (53%), and ataxia (42%). We conclude that TD is underdiagnosed in this population with consequent morbidity and mortality. TD likely develops because of inflammatory stress from CIC’s compounded by AIC’s combined with decreased energy intake or increased nutrient losses.

## 1. Introduction

Thiamine micronutrient deficiency (TD) can cause a variety of non-specific symptoms and leads to a variety of thiamine deficiency disorders (TDDs) [1,2] such as heart failure, type 2 lactic acidosis, polyneuropathy, Wernicke’s Encephalopathy (WE) and Korsakoff syndrome [1,2,3]. Left untreated, more severe disorders can lead to permanent disability or death. TD and TDDs are reportedly rare in high income countries except in alcohol abusers (2.8% or less) although there is a lack of prevalence data [4]. Symptoms are often vague and non-specific such as fatigue, leg edema, imbalance, confusion, mood disorders, gastrointestinal (GI) upset, and muscle weakness [1] which likely contributes to under diagnosis of TDDs in non-alcoholics. There is literature to suggest it is not rare in the acutely and chronically ill in developed countries where food security is common [3,5,6,7,8].

The body’s supply of thiamine depends entirely on dietary intake, there is no endogenous synthesis [1,3]. Thiamine diphosphate is an essential cofactor for the metabolism of carbohydrates and amino acids [1,3]. TD can develop quickly, within 18 days on a thiamine deficient diet or within 72 h in critically ill patients [9]. Any disease that adversely affects intake or absorption, accentuates loss of nutrients, or causes acute inflammatory stress can lead to development of TD in a matter of days. There are many restrictive diets which minimize the intake of thiamine fortified foods such as wheat flour (a gluten free diet for example) that could lead to TD. The high prevalence of TD in obese patients who are dieting suggests even typical weight loss diets predispose to its development [10].

There have been many published accounts of TD in acutely and chronically ill patients with conditions such as end stage renal disease [11], cancer [12,13,14], heart failure [15,16,17,18], dementia [19,20], acute psychiatric illness [21], stroke [22], diabetic ketoacidosis [23], critical illness [2], and medically complicated obesity [10,24]. Acute Illnesses which increase metabolic demands can lead to TD more quickly including severe sepsis [2,9]. Elderly patients admitted to hospital seem particularly vulnerable [5,7,8]. Despite a preponderance of evidence that TD is not rare in these populations, it is still under-diagnosed and under-treated [4].

We hypothesize that TD is not rare in hospitalized patients who generally have higher chronic disease burden [25] and an incidence of malnutrition of 24% or more [26,27,28]. The etiologies of TD can be broadly categorized as insufficient intake (including malabsorption and toxin interference), increased need due to inflammatory stress (e.g., sepsis), and increased losses (such as vomiting, diarrhea, dialysis [29], or chronic diuretic use [16]). Hospitalized patients often have increased inflammatory stress due to acute and chronic forms of inflammation with concomitant cachexia [30,31]. Many use diuretics for hypertension and heart failure treatment. The purpose of this study is to describe a series of 36 cases of TD in hospitalized non-alcoholic patients in a Veterans Affairs (VA) hospital, defined by plasma thiamine level less than or equal to 7 nmol/L. We describe their symptoms of TDD’s as well as possible underlying factors leading to TD. Our overall goal is to generate data which help to define the problem of TD in hospitalized veterans and lead to future efforts on mitigation.

## 2. Materials and Methods

This is a retrospective case series of hospitalized non-alcoholics at the VA hospital in Reno, NV who were identified as having TD defined as abnormally low plasma thiamine concentration. In most cases the thiamine level was ordered because of suggestive symptoms. Cases were identified in two ways: by personal knowledge of author EM, and by querying the laboratory database of the VA Sierra Nevada Healthcare System (VASNHCS) from 9 December 2014 to 3 September 2020 for values of 7 nmol/L or less. Plasma thiamine measurements were performed at Quest Diagnostics in Valencia, California using Liquid Chromatography/Tandem Mass Spectrometry with a normal range of 8–30 nmol/L. Cases were excluded if there was a history of unhealthy alcohol use as described by The National Institute on Alcohol Abuse and Alcoholism (more than 14 standard drinks per week for men less than age 65, or more than 7 per week for women or men 65 years or older). Additionally, cases were excluded if they were not hospitalized within +/− 4 weeks of the result. Exclusions were determined by chart review. The laboratory database query returned 84 individuals with low thiamine levels. Of those, sixteen were excluded due to excess alcohol intake and thirty-five were excluded for not having been hospitalized within four weeks of the result. Three additional cases were identified in the clinical practice of author EM for a total of thirty-six cases.

All patient data including demographics, medical history, and laboratory values were extracted from their electronic medical records at VASNHCS. This study was reviewed by the University of Nevada, Reno Institutional Review Board (IRB) and determined to be exempt from IRB review under Category 4, secondary research utilizing identifiable private information recorded by the investigator in such a manner that the identity of the human subjects cannot be readily ascertained.

Clinical and laboratory data were extracted within +/− 4 weeks of the abnormally low thiamine result. For non-thiamine tests with multiple results within that timeframe, the value closest to the date and time of the thiamine test was chosen. Demographic data included age on the date of the thiamine result, sex assigned at birth, race, and date of death where applicable. Anthropometric data include actual standing or bed weight on admission, trend in weight in the preceding weeks or months (within 1 year) as a percentage of their baseline weight (average weight before the onset of weight loss), and body mass index (BMI) at the time of admission. We extracted albumin (g/dL) and prealbumin (mg/dL) as biomarkers of malnutrition and acute inflammation [32]. We noted magnesium deficiency, lactic acid (mmol/L), alcohol level (mg/dL) and urine drug screens where available. We recorded other micronutrient deficiencies including vitamin C, D, B12, folate, and iron.

Chart notes were reviewed for acute medical conditions diagnosed during hospitalization and chronic medical conditions present before admission. Causes of increased thiamine loss (IL) were defined as nausea, vomiting, diarrhea, diuretic use, or dialysis. Inflammatory stress (IS) was defined as any acute or chronic inflammatory condition. Insufficient intake (II) was determined from dietician, provider, and case management or social work notes. A condition was considered to cause acute inflammation if it was due to active cancer, major burns, acute crystal-induced arthropathy, untreated acute autoimmune disease, major infection, acute pancreatitis, recent major surgery, tissue infarction, or trauma [33,34]. Conditions considered to cause chronic inflammation included stable autoimmune disease on therapy, congestive heart failure (CHF), chronic obstructive pulmonary disease (COPD), cancer in remission (not cured), chronic kidney disease (CKD) with dialysis status, atherosclerotic cardiovascular disease (ASCVD), diabetes mellitus (DM), cirrhosis of the liver, obesity (BMI > 24.9), tobacco abuse, and chronic infection [34]. A diagnosis of malignancy or autoimmune disease was only included if confirmed by biopsy or subspecialist consultant opinion.

Cases were reviewed for signs and symptoms of TD as described in nursing, physician, and allied health professional’s notes. A symptom was only listed as due to TD if there were no other more likely explanations for it. For example, peripheral or pulmonary edema were not listed if there was a prior history of edema, congestive heart failure, renal, or liver failure. GI distress was not listed if there was evidence of an acute gastrointestinal illness caused by other defined etiologies. Peripheral neuropathy was not listed if there was a prior history of it.

Current substance abuse or abnormal drug screens were noted. Active medications prior to admission were evaluated with the rationale that they were more likely to have influenced development of TD than those given acutely in the hospital. Dietician consults were reviewed for information on sufficiency of energy intake, specialized diets, social and medical factors influencing diet, and physical findings of muscle and fat loss. Functional status was determined to be reduced if there was a history of weakness or falling or if loss of function was noted on physical or occupational therapist evaluations.

Echocardiogram results within 4 weeks of the abnormal thiamine level were noted and compared to any prior results. Specialty consults and neuroimaging completed for symptoms related to TD were recorded as well as discharge disposition as markers of increased cost of care. We tabulated whether cases were treated with thiamine along with what dose.

## 3. Results

Table 1 lists risk factors for developing TD and the percentages of cases with each risk factor. Table S1 lists demographics of each case, acute diagnoses treated in the hospital, a count of each of their acute and chronic inflammatory conditions (AICs and CICs, respectively), probable causes of TD, and whether they are deceased. The average age was 75.4 years (median 75, range 61–91) and most were male (3 females), reflecting our low census of female inpatients. Eighty-three percent were white, there was one African American, one American Indian or Alaska Native, one native Hawaiian or other Pacific Islander, and two of mixed race. The diagnoses treated during hospitalization were wide ranging but 39% had concomitant adult failure to thrive (FTT) and 44% had moderate or severe protein calorie malnutrition (PCM). Six had no AICs, twenty-three had one AIC, and seven had two AICs. All had at least one CIC, 97% had two or more. Sixty four percent had two probable causes of TD, 33% had 3 likely causes, and one case had one precipitating factor. All had inflammatory stress in the form of acute or chronic inflammatory conditions. Nineteen (53%) were deceased at the time of chart review, one is lost to follow-up. Two deaths occurred within a week of the low thiamine level, seven more within 90 days.

**Figure 1:**
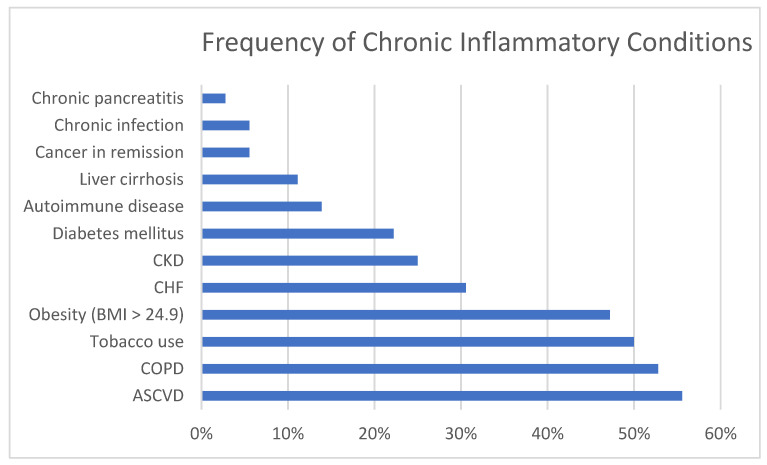
Percentage of cases with specific chronic inflammatory conditions. CKD = Chronic kidney disease; CHF = Congestive heart failure; COPD = Chronic obstructive pulmonary disease; ASCVD = atherosclerotic cardiovascular disease.

**Figure 2:**
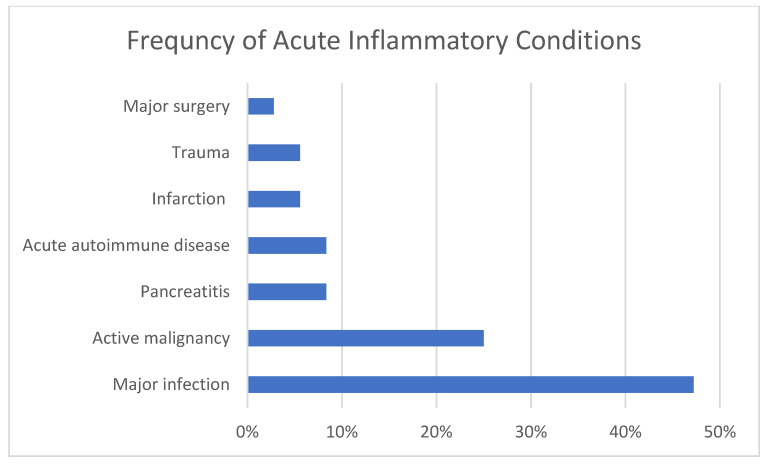
Percentage of cases with acute inflammatory conditions.

Figure 1 illustrates the percentage of cases with specific CICs and Figure 2 the percentage of cases with categories of AICs. Of the causes of chronic inflammation, ASCVD, COPD, tobacco use, and obesity were most prevalent, followed by CHF, CKD (one on dialysis), and DM. Less common were stable autoimmune disease, cirrhosis, cancer in remission, chronic infection, and chronic pancreatitis. The most common causes of acute inflammation were systemic infections and acute malignancy, followed by pancreatitis and acute autoimmune diseases. Tissue infarction, major surgery and trauma were less common although we do not admit many cases of trauma to our facility. Two of the patients had previously undergone bariatric surgery which is known to predispose to TD [10,24].

Many of the cases were found to have insufficient energy/dietary intake. Seventeen percent reported homelessness or food insecurity, 14% were from a skilled nursing facility or assisted living, and 33% reported reduced appetite due to their underlying medical problems. Detailed dietary information was not available. Of the fifteen who had drug and alcohol laboratory screens, one was positive for methamphetamine, one reported regular marijuana use, and none had detectable levels of alcohol, so substance abuse was not a contributing factor.

Table 2 lists the frequency of reported signs of malnutrition. Table S2 illustrates individual case data on body mass index (BMI) in kg/m^2^, trend in weight loss, biomarkers of malnutrition and inflammation, and associated signs of malnutrition as defined by the American Society for Parenteral and Enteral Nutrition (ASPEN) [35]. There was a wide variation in BMI from 15.8 to 43.7, 47% were overweight or obese (BMI > 24.9) with 14% being underweight (BMI < 18.5). Seventy-nine percent had significant weight loss (5% or more) in the preceding months, 14% had no weight change, one gained 4%, and two had inaccurate weights. Seventy-two percent had an abnormally low albumin (<3.5 g/dL) and in 11 cases where prealbumin was measured, all were low (<18 mg/dL). Thirty-one percent had low magnesium (<1.8 mg/dL), a necessary cofactor in thiamine metabolism [3]. There were twenty-eight nutrition consults with 89% deemed to have insufficient energy intake. In 33 cases functional status was assessed 82% were determined to have reduced functional ability with eight so disabled they were no longer able to ambulate. Of nineteen cases where information was extractable, 95% showed fat or muscle loss. Thirty-one cases had data on other micronutrient deficiencies of which 47% were deficient in one or more of iron, vitamin C, vitamin B12 (four cases), folate (one case), or vitamin D (6 cases). These data suggest that assessments set forth by ASPEN are good predictors of risk for TD when taken together with recent weight loss, low albumin and prealbumin [35].

Table 3 shows the frequency of signs and symptoms of TD in the 36 cases with 92% having at least one symptom. Table S3 shows individual case data of each sign and symptom. Weakness and falling were the most prevalent at 75%, followed by neuropsychiatric manifestations at 72%, (encephalopathy or delirium, delusions, hallucinations, agitation, odd behaviors, personality change, or seizures), and GI symptoms at 53% (anorexia, nausea and vomiting, gastroparesis, ileus, or constipation). Forty-two percent exhibited ataxia when we included cases described by physical or occupational therapists as unsteady gait. While only four had documented ophthalmoplegia, we noted that this exam finding was poorly documented. Four cases met the triad of signs defining WE (delirium, ataxia, and ophthalmoplegia). Twenty-nine (81%) met the Caine criteria [36] for WE with 2 or more of nutritional deficiency (low thiamine level), abnormal mental status, cerebellar dysfunction, or ophthalmoplegia. Twenty-two percent had symptoms of wet beriberi with new non-ischemic cardiomyopathy, pulmonary edema, or peripheral edema although many cases with these findings were excluded due to possible alternate explanations. Fourteen percent had new and otherwise unexplained peripheral neuropathy. Four exhibited lactic acidosis but those were associated with more likely explanations such as sepsis and liver failure.

With respect to medication use prior to admission, 78% were prescribed nutritional supplements defined as vitamins, minerals, or nutritional drinks. Fifty-three percent were prescribed pain relievers, 53% an angiotensin-converting enzyme inhibitor or angiotensin receptor blocker, 47% a beta blocker, 39% a proton pump inhibitor, statin, or anti-platelet agent, 36% an alpha blocker, 31% a psychotropic medication, and 28% were taking a diuretic. The average number of medications and supplements prescribed was 10.9 with a range of 0 to 33 per individual prompting us to wonder if polypharmacy contributes to loss of appetite.

Seventeen cases (47%) prompted specialty consultation for symptoms most likely due to TD, most frequently mental health followed by neurology, geriatrics, and physical medicine and rehabilitation. Fifty percent had CT or MRI of their brain and/or spinal cord looking for central nervous system lesions that would explain weakness or neuropsychiatric symptoms or because falls resulted in trauma. Nine had one imaging study, seven had two, and two had four or more studies. Of the studies completed, only one showed an acute change with extension of a prior stroke. The rest were normal, showed old previously known pathology, or non-specific changes. Four had recurrent admissions to the hospital for delirium and failure to thrive. Of those admitted from home, eleven (31%) were discharged to a skilled nursing facility (SNF), nine (25%) went home with home health therapy needs, five (14%) were discharged on hospice, and one died in the hospital.

Of the 36 cases, 25 were prescribed thiamine but the dose and route were variable ranging 100 mg orally per day to 500 mg parenterally three times a day. Due to the retrospective nature of this case series, it was impossible to determine if symptoms improved with therapy in all cases. In cases in which we participated in the care, most improved with IV or IM supplementation and some had complete symptom resolution.

## 4. Discussion

This is the largest case series of TD in non-alcoholic hospitalized veterans to date and it complements many published series of TD in acutely and chronically ill adults as described in the introduction. Due to the integrated VA healthcare system where most veterans get nearly all their healthcare and social services support, we were well suited to describe the pre-TD longitudinal data and potential underlying factors. Taken together with prior published reports, our findings suggest TD in hospitalized patients is far more prevalent than has been traditionally considered, review of the literature suggests it is likely between 10 and 25% [5,6,7,8,9,17,19,20,22,37].

The clinical presentation of TD in non-alcoholics appears to differ from that in alcoholics in that weakness and falling are frequent symptoms (also noted by Pepersack et al. [8]) and gastrointestinal manifestations were common. Chamorro et al. also note differences in presentation between alcoholic and non-alcoholic patients with WE [38]. Many of our veterans presented with psychiatric symptoms such as delusions, hallucinations, or personality changes, as well as typical delirium. Concomitant diagnoses of adult failure to thrive and moderate to severe protein calorie malnutrition were common and should prompt hospital providers to consider TD. Edema and non-ischemic heart failure were less frequent but significant in that the etiology of such were labelled idiopathic without consideration of TD. Falls and delirium are common and important complications in hospital medicine and TD should be considered in the differential diagnosis of their etiology, with empiric treatment strongly recommended. Forty-seven percent of our cases had additional micronutrient deficiencies which may have contributed to their symptom complex. Micronutrient deficiencies have been linked to common and non-specific syndromes such as migraine headaches [39], weakness and frailty [40,41,42], delirium [43], and polyneuropathy [44] and should be considered in the differential diagnosis of these symptoms along with TD.

The inflammatory stress of several common chronic diseases are likely important risk factors for development of TD with 100% of our cases having at least one CIC, 97% with two or more. It is probable that AICs such as infection or untreated cancer precipitate TD in patients with CICs whose thiamine stores are chronically stressed. Many of these veterans had poor diet due to appetite or financial limitations and ironically TD accentuates poor intake by causing ileus and gastroparesis. This precipitates a downward spiral of insufficient intake and ongoing inflammatory stress. If there are also increased losses due to nausea, vomiting, diarrhea, dialysis, or diuretic use, the risk of developing TD may be increased.

The measurement of thiamine is typically done at specialized reference laboratories which results in delays in obtaining results. This presents a significant diagnostic challenge, especially when most hospital systems aim for discharging patients in a timely manner. This likely led to our provider’s reluctance to definitively document WE despite meeting the Caine criteria and empirically treating with thiamine replacement. Our results support the use of recent weight loss, physical findings of muscle and fat wasting, reduced functional status and low albumin or pre-albumin to suspect TD is present. TD did not seem to correlate with other B vitamin deficiencies and prescribing vitamins and dietary supplements did not appear to prevent its development.

TD not only causes morbidity and mortality, but it is also an expensive problem. Many of these individuals had neuroimaging to look for etiology of weakness, delirium, or other neurologic symptoms or because of trauma workup from falling, the vast majority of which showed no acute abnormalities. Many had specialty consults to assist in diagnosis. Even more significant is the number who required transfer to a skilled nursing facility or in home care due to inability to care for themselves in the community.

While we do not conclude the observed deaths in this case series were due to TD itself, it is possible that this underlying condition may have complemented the acute and chronic medical conditions and advanced age of the patients to increase risk of mortality. This should be an area of future study. Development of TD should be considered a marker of the severity of the underlying illness and should prompt practitioners to intensify efforts at treating those illnesses or pursuing palliative care if they are untreatable. Even if those illnesses are not curable, treating delirium due to TD may result in improved quality of life with more meaningful interactions with family.

There is no clear consensus on which dose and route is necessary to treat symptoms of TD and as such, there is wide variation in the method of thiamine repletion in clinical practice. The appropriate dose, route, and frequency of thiamine replacement for various TDD’s is a topic that would benefit from randomized controlled clinical trials. It is generally accepted that 500 mg IV every 8 h is sufficient to treat WE, but it is suspected far less is necessary to treat other TDD’s.

One of the limitations of this study was the preponderance of men (92%) and white race (83%), due to the typical makeup of our older veteran population in Reno, NV. Another limitation is the availability of optimized thiamine bioassays. VASNHCS uses plasma thiamine level, which measures free thiamine and thiamine monophosphate most reflective of recent nutrient intake [1]. Measurement of whole blood thiamine reflects total body thiamine stores. Regardless of which assay is used, there is a lack of data correlating thiamine levels to clinically relevant symptoms of TD as pointed out by Whitfield et al. [1]. Many studies of TD report different cutoff values for abnormally low thiamine making it difficult to compare results. The historical functional assay used to define TD, the erythrocyte transketolase (ETK) assay, is no longer available in the United States and has been supplanted by direct measurement of thiamine in plasma or whole blood. This makes it difficult to compare more recent studies of TD to older studies which define TD by the results of the ETK biologic assay. Point of care (POC) measurement of thiamine is possible but has not been developed commercially [1]. A POC test would be highly beneficial in inpatient settings for rapid diagnosis.

## 5. Conclusions

This case series brings to light the importance of considering TD in the differential diagnosis of delirium, weakness and falling as well as other common symptoms in malnourished hospitalized elderly veterans with considerable inflammatory burden. These cases highlight the typical and atypical symptoms of TDD’s and draws a connection between the development of TD and inflammatory stress. Clinicians should be aware of trends in weight loss, functional status, evidence of muscle and fat loss, and correlate with low albumin and prealbumin as well as acute and chronic inflammatory conditions to determine if TD should be considered. Empiric treatment with IV or IM thiamine is inexpensive and well tolerated and could improve outcomes in this population. This case series underscores the need for further investigation, namely prospectively testing malnourished non-alcoholic hospitalized patients with reduced functional capacity and inflammatory stress or accentuated losses for thiamine levels and correlating with symptoms of TD to determine prevalence of TD in this population. Understanding the social and nutritional determinants of health that are contributing to this problem in veterans would also be important.

## Figures and Tables

**Table 1:** Risk factors for developing TD.

Risk Factor	Number (%)
Insufficient intake	28 (78%)
Inflammatory stress	36 (100%)
0 Chronic inflammatory condition	0
1 Chronic inflammatory condition	1 (2.7%)
2 Chronic inflammatory conditions	10 (27.8%)
3 Chronic inflammatory conditions	10 (27.8%)
4 Chronic inflammatory conditions	9 (25%)
5 Chronic inflammatory conditions	6 (16.7%)
0 Acute inflammatory condition	6 (16.7%)
1 Acute inflammatory condition	23 (63.9%)
2 Acute inflammatory conditions	7 (19.4%)
Increased losses	19 (52.8%)

**Table 2:** Associated signs of malnutrition.

Sign	Number of Cases with Data	Number (%)
BMI less than 18.5	36	5 (14%)
Non-volitional weight loss ≥ 5% of baseline ^1^	34	27 (79%)
Albumin less than 3.5 g/dL	36	26 (72%)
Prealbumin less than 18 mg/dL	11	11 (100%)
Reduced energy intake ^2^	28	25 (89%)
Reduced functional status ^3^	33	27 (82%)
Physical findings of muscle or fat loss ^4^	19	18 (95%)

^1^ Average weight in the preceding 6–12 months; ^2^ As determined by dietician consult opinion; ^3^ Determined by history of weakness or falling or by physical or occupational therapist consult opinion; ^4^ Based on dietician or physician physical assessment.

**Table 3:** Signs and symptoms of TD.

Symptom	Number (%)
Weakness or falling	27 (75%)
Neuropsychiatric symptoms	26 (72%)
Gastrointestinal symptoms	19 (53%)
Ataxia	15 (42%)
Polyneuritis	5 (14%)
Peripheral or pulmonary edema	5 (14%)
Ophthalmoplegia	4 (11%)
Non-ischemic congestive heart failure	3 (8%)

## Data Availability

The data presented in this study are available on request from the corresponding author. The data are not publicly available due to privacy.

## Supplementary Materials

The following are available online at https://www.mdpi.com/article/10.3390/jcm10071449/s1, Table S1: Patient demographics, acute medical conditions treated in the hospital, number of acute and chronic inflammatory conditions, proposed mechanisms of TD, and mortality, Table S2: BMI, weight change, biomarkers of malnutrition and inflammation, magnesium deficiency, and associated signs of malnutrition, Table S3: Signs and symptoms of thiamine deficiency.

## References

[1] Whitfield K.C., Bourassa M.W., Adamolekun B., Bergeron G., Bettendorff L., Brown K.H., Cox L., Fattal-Valevski A., Fischer P.R., Frank E.L. (2018). Thiamine Deficiency Disorders: Diagnosis, Prevalence, and a Roadmap for Global Control Programs. Ann. N. Y. Acad. Sci..

[2] Attaluri P., Castillo A., Edriss H., Nugent K. (2018). Thiamine Deficiency: An Important Consideration in Critically Ill Patients. Am. J. Med. Sci..

[3] Lonsdale D., Michael Eskin N.A. (2018). Thiamin. Advances in Food and Nutrition Research.

[4] Galvin R., Bråthen G., Ivashynka A., Hillbom M., Tanasescu R., Leone M.A. (2010). EFNS Guidelines for Diagnosis, Therapy and Prevention of Wernicke Encephalopathy. Eur. J. Neurol..

[5] Lee D.C., Chu J., Satz W., Silbergleit R. (2000). Low Plasma Thiamine Levels in Elder Patients Admitted through the Emergency Department. Acad. Emerg. Med..

[6] Lemoine A., Le Devehat C., Codaccioni J.L. (1980). Vitamin B1, B2, B6, and C Status in Hospital Inpatients. Am. J. Clin. Nutr..

[7] O’Keeffe S.T., Tormey W.P., Glasgow R., Lavan J.N. (1994). Thiamine Deficiency in Hospitalized Elderly Patients. Gerontology.

[8] Pepersack T., Garbusinski J., Robberecht J., Beyer I., Willems D., Fuss M. (1999). Clinical Relevance of Thiamine Status amongst Hospitalized Elderly Patients. Gerontology.

[9] Donnino M.W., Carney E., Cocchi M.N., Barbash I., Chase M., Joyce N., Chou P.P., Ngo L. (2010). Thiamine Deficiency in Critically Ill Patients with Sepsis. J. Crit. Care.

[10] Nath A., Tran T., Shope T.R., Koch T.R. (2017). Prevalence of Clinical Thiamine Deficiency in Individuals with Medically Complicated Obesity. Nutr. Res..

[11] Hung S.C., Hung S.H., Tarng D.C., Yang W.C., Chen T.W., Huang T.P. (2001). Thiamine Deficiency and Unexplained Encephalopathy in Hemodialysis and Peritoneal Dialysis Patients. Am. J. Kidney Dis..

[12] Bleggi-Torres L.F., De Medeiros B.C., Werner B., Zanis Neto J., Loddo G., Pasquini R., De Medeiros C.R. (2000). Neuropathological Findings after Bone Marrow Transplantation: An Autopsy Study of 180 Cases. Bone Marrow Transplant..

[13] Isenberg-Grzeda E., Alici Y., Hatzoglou V., Nelson C., Breitbart W. (2016). Nonalcoholic Thiamine-Related Encephalopathy (Wernicke-Korsakoff Syndrome) Among Inpatients with Cancer: A Series of 18 Cases. Psychosomatics.

[14] Levavi H., Park D., Tannenbaum J., Steinberg A. (2019). Retrospective Analysis of Thiamine Deficiency in Allogeneic Stem Cell Transplant Patients. Ann. Hematol..

[15] Abou-Hashem R.M., Maamoun M.M.A., Hamza S.A., Fahmy H.M., Mortagy A.K. (2009). Thiamine Level in Hospitalized Elderly Egyptian Patients with Congestive Heart Failure and Left Ventricular Systolic Dysfunction. J. Am. Geriatr. Soc..

[16] Brady J.A., Rock C.L., Horneffer M.R. (1995). Thiamin Status, Diuretic Medications, and the Management of Congestive Heart Failure. J. Am. Diet. Assoc..

[17] Hanninen S.A., Darling P.B., Sole M.J., Barr A., Keith M.E. (2006). The Prevalence of Thiamin Deficiency in Hospitalized Patients with Congestive Heart Failure. J. Am. Coll. Cardiol..

[18] Jain A., Mehta R., Al-Ani M., Hill J.A., Winchester D.E. (2015). Determining the Role of Thiamine Deficiency in Systolic Heart Failure: A Meta-Analysis and Systematic Review. J. Card. Fail..

[19] Gold M., Chen M., Johnson K. (1995). Plasma and Red Blood Cell Thiamine Deficiency in Patients with Dementia of the Alzheimer’s Type. Arch. Neurol..

[20] Vognar L., Stoukides J. (2009). The Role of Low Plasma Thiamin Levels in Cognitively Impaired Elderly Patients Presenting with Acute Behavioral Disturbances. J. Am. Geriatr. Soc..

[21] Lin S., Leppla I.E., Yan H., Probert J.M., Randhawa P.A., Leoutsakos J.M.S., Probasco J.C., Neufeld K.J. (2020). Prevalence and Improvement of Caine-Positive Wernicke-Korsakoff Syndrome in Psychiatric Inpatient Admissions. Psychosomatics.

[22] Ehsanian R., Anderson S., Schneider B., Kennedy D., Mansourian V. (2020). Prevalence of Low Plasma Vitamin B1 in the Stroke Population Admitted to Acute Inpatient Rehabilitation. Nutrients.

[23] Moskowitz A., Graver A., Giberson T., Berg K., Liu X., Uber A., Gautam S., Donnino M.W. (2014). The Relationship between Lactate and Thiamine Levels in Patients with Diabetic Ketoacidosis. J. Crit. Care.

[24] Shah H.N., Bal B.S., Finelli F.C., Koch T.R. (2013). Constipation in Patients with Thiamine Deficiency after Roux-En-Y Gastric Bypass Surgery. Digestion.

[25] Zulman D.M., Chee C.P., Wagner T.H., Yoon J., Cohen D.M., Holmes T.H., Ritchie C., Asch S.M. (2015). Multimorbidity and Healthcare Utilisation among High-Cost Patients in the US Veterans Affairs Health Care System. BMJ Open.

[26] de Luis D., Lopez Guzman A. (2006). Nutritional Status of Adult Patients Admitted to Internal Medicine Departments in Public Hospitals in Castilla y Leon, Spain—A Multi-Center Study. Eur. J. Intern. Med..

[27] Kaiser M.J., Bauer J.M., Rämsch C., Uter W., Guigoz Y., Cederholm T., Thomas D.R., Anthony P.S., Charlton K.E., Maggio M. (2010). Frequency of Malnutrition in Older Adults: A Multinational Perspective Using the Mini Nutritional Assessment. J. Am. Geriatr. Soc..

[28] Win A.Z., Ceresa C., Arnold K., Allison T.A. (2017). High Prevalence of Malnutrition among Elderly Veterans in Home Based Primary Care. J. Nutr. Heal. Aging.

[29] Dizdar O.S., Yıldız A., Gul C.B., Gunal A.I., Ersoy A., Gundogan K. (2020). The Effect of Hemodialysis, Peritoneal Dialysis and Renal Transplantation on Nutritional Status and Serum Micronutrient Levels in Patients with End-Stage Renal Disease; Multicenter, 6-Month Period, Longitudinal Study. J. Trace Elem. Med. Biol..

[30] Evans W.J., Morley J.E., Argilés J., Bales C., Baracos V., Guttridge D., Jatoi A., Kalantar-Zadeh K., Lochs H., Mantovani G. (2008). Cachexia: A New Definition. Clin. Nutr..

[31] Jensen G.L., Mirtallo J., Compher C., Dhaliwal R., Forbes A., Figueredo Grijalba R., Hardy G., Kondrup J., Labadarios D., Nyulasi I. (2010). Adult Starvation and Disease-Related Malnutrition: A Proposal for Etiology-Based Diagnosis in the Clinical Practice Setting from the International Consensus Guideline Committee. J. Parenter. Enter. Nutr..

[32] Keller U. (2019). Nutritional Laboratory Markers in Malnutrition. J. Clin. Med..

[33] Gabay C., Kushner I. (1999). Acute-Phase Proteins and Other Systemic Responses to Inflammation. N. Engl. J. Med..

[34] Pahwa R., Jialal I. (2019). Chronic Inflammation—StatPearls—NCBI Bookshelf.

[35] White J.V., Guenter P., Jensen G., Malone A., Schofield M. (2012). Consensus Statement: Academy of Nutrition and Dietetics and American Society for Parenteral and Enteral Nutrition: Characteristics Recommended for the Identification and Documentation of Adult Malnutrition (Undernutrition). J. Parenter. Enter. Nutr..

[36] Caine D., Halliday G.M., Kril J.J., Harper C.G. (1997). Operational Criteria for the Classification of Chronic Alcoholics: Identification of Wernicke’s Encephalopathy. J. Neurol. Neurosurg. Psychiatry.

[37] Jamieson C.P., Obeid O.A., Powell-Tuck J. (1999). The Thiamin, Riboflavin and Pyridoxine Status of Patients on Emergency Admission to Hospital. Clin. Nutr..

[38] Chamorro A.J., Rosón-Hernández B., Medina-García J.A., Muga-Bustamante R., Fernández-Solá J., Martín-González M.C., Seco-Hernández E., Novo-Veleiro I., Suárez-Cuervo C., Mateos-Díaz A.M. (2017). Differences Between Alcoholic and Nonalcoholic Patients With Wernicke Encephalopathy: A Multicenter Observational Study. Mayo Clin. Proc..

[39] Liampas I., Siokas V., Mentis A.A., Aloizou A., Dastamani M., Tsouris Z., Aslanidou P., Brotis A., Dardiotis E. (2020). Serum Homocysteine, Pyridoxine, Folate, and Vitamin B12 Levels in Migraine: Systematic Review and Meta-Analysis. Headache J. Head Face Pain.

[40] Soh Y., Won C.W. (2020). Association between Frailty and Vitamin B12 in the Older Korean Population. Medicine.

[41] van den Berg K.S., Arts M.H.L., Collard R.M., van den Brink R.H.S., Comijs H.C., Marijnissen R.M., Oude Voshaar R.C. (2020). Vitamin D Deficiency and Course of Frailty in a Depressed Older Population. Aging Ment. Health.

[42] Zhou J., Huang P., Liu P., Hao Q., Chen S., Dong B., Wang J. (2016). Association of Vitamin D Deficiency and Frailty: A Systematic Review and Meta-Analysis. Maturitas.

[43] Sanford A.M., Flaherty J.H. (2014). Do Nutrients Play a Role in Delirium?. Curr. Opin. Clin. Nutr. Metab. Care.

[44] Yang G.T., Zhao H.Y., Kong Y., Sun N.N., Dong A.Q. (2018). Correlation between Serum Vitamin B12 Level and Peripheral Neuropathy in Atrophic Gastritis. World J. Gastroenterol..

