# Supplementary material for: A Retrospective Case Series of Thiamine Deficiency in Non-Alcoholic Hospitalized Veterans: An Important Cause of Delirium and Falling?
Source: J Clin Med. 2021 Apr 1;10(7):1449. doi: 10.3390/jcm10071449 (PMC8037750; doi:10.3390/jcm10071449)
Supplement: Supplementary file 1 [file jcm-10-01449-s001.pdf]

Table S1. Patient demographics, acute medical conditions treated in the hospital, number of acute and chronic inflammatory conditions, proposed mechanisms of TD, and mortality.

Case	Age/G	Race	Acute Diagnoses Treated During Hospitalization	#AICs	#CICs	Proposed Mechanisms of TD[2]	Deceased
1	87 M	White	Acute on chronic hyponatremia, stage IV melanoma on checkpoint inhibiter therapy, adult FTT, encephalopathy, severe PCM	1	1	II, IS, IL	Yes
2	73 M	AA	Pneumonia, acute kidney injury, systolic CHF exacerbation, Mobitz II heart block, severe PCM	1	4	II, IS, IL	No
3	75 F	White	Encephalopathy, weakness & falling, adult FTT, acute kidney injury, metabolic acidosis, diarrhea, UTI, polypharmacy	1	3	II, IS, IL	Yes
4	86 M	White	Aspiration pneumonia with sepsis, adult FTT, encephalopathy, moderate PCM	1	2	II, IS, IL	Yes
5	61 M	White	Acute pancreatitis, hypomagnesemia, hypophosphatemia, moderate PCM, methamphetamine abuse	1	2	II, IS	UA
6	84 M	White	Encephalopathy, bullous skin rash, weakness & falls, hypercalcemia, dehydration, UTI, dysphagia, adult FTT, decubitus ulcer, severe PCM	2	2	II, IS	Yes
7	65 M	White	Encephalopathy, weakness, adult FTT, urinary retention, dehydration, AKI, moderate PCM, multiple electrolyte deficiencies, CLL on anti-CD20 treatment	1	4	II, IS	Yes
8	63 M	White	Wernicke's encephalopathy, ophthalmoplegia, weakness & falling, pancreatitis, moderate PCM, folate deficiency, hypomagnesemia, coumadin toxicity	1	3	II, IS	No
9	75 M	White	Cholecystitis, AKI, encephalopathy, renal infarction	2	5	IS, IL	Yes
10	71 M	White	Recurrent N/V due to idiopathic gastroparesis, non-infectious diarrhea, new onset AFIB with RVR, weakness & falling, severe PCM with starvation ketoacidosis, dehydration, AKI, hypokalemia, hypomagnesemia, adult FTT	0	4	II, IS, IL	No
11	89 M	White	Acute on chronic anemia of unclear etiology, acute hypoxic RF, new onset CHF with reduced EF, pulmonary AVM, weakness, dizziness, adult FTT	1	2	II, IS	Yes
12	76 M	White	Pneumonia with sepsis & hypoxic respiratory failure, encephalopathy, AKI, urinary retention, acute on chronic abdominal pain, ileus	1	5	II, IS, IL	Yes
13	90 M	White	Pneumonia, AFIB w RVR, HIT, weakness, adult FTT, severe PCM	2	5	II, IS, IL	Yes
14	80 M	NH/PI	Acute stroke, untreated polycythemia vera	2	4	IS, IL	No
15	84 M	White	UTI w sepsis, urinary obstruction, acute kidney injury	1	3	II, IS	Yes
16	70 M	White	Complications of Parkinson's including auditory & visual hallucinations, agitation, delirium, dysphagia with aspiration pneumonia & sepsis,	1	3	IS, IL	Yes

			hypoxic RF, upper GI bleeding, autonomic insufficiency, acute kidney injury				
17	77 M	White	Non-infectious diarrhea, weakness & falling, adult FTT, hypertensive urgency, dehydration, hypomagnesemia	0	3	II, IS, IL	No
18	74 M	White	Acute pathologic compression fractures of spine, encephalopathy, acute multiple myeloma, anemia of neoplastic disease, hypercalcemia, AKI, N/V, hypomagnesemia	1	4	IS, IL	No
19	70 M	White	Pneumonia, COPD exacerbation w acute hypoxic respiratory failure, hypomagnesemia	1	4	II, IS, IL	No
20	64 M	White	Post-operative encephalopathy & psychosis, hypertensive urgency, hemi-colectomy for large tubular adenomas, hypokalemia.	1	2	II, IS	No
21	81 M	White	AFIB w RVR, weakness & falling	0	5	II, IS	No
22	86 M	White	Weakness & falling, rhabdomyolysis, AFIB with bradycardia requiring pacemaker, poorly defined autoimmune process with new elevated ANA	1	2	IS, IL	No
23	69 M	White	Weakness, orthostatic hypotension, N/V, AKI, hyponatremia, adult FTT, severe PCM, hospital acquired UTI with sepsis	1	4	II, IS, IL	Yes
24	91 M	White	Acute mastoiditis, encephalopathy, weakness and falling, wrist fracture, non-sustained VT, NSTEMI type 2, severe PCM, invasive squamous cell carcinoma of face.	2	2	II, IS	Yes
25	78 M	AI/AK	New onset cirrhosis with portal hypertension, ascites, lactic acidosis, non-infectious diarrhea, severe PCM	0	3	II, IS	Yes
26	69 M	UA	Spontaneous bacterial peritonitis, sepsis, cirrhosis with ascites, nausea and vomiting	1	2	IS, IL	Yes
27	74 M	White	Acute pancreatitis w pancreatic pseudocyst.	1	4	IS	Yes
28	78 M	White	B cell lymphoma admitted for biopsy and initiation of chemotherapy, hypercalcemia, acute kidney injury, encephalopathy, non-sustained ventricular tachycardia, hyponatremia	1	2	IS, IL	No
29	79 M	White & AI/AK	Acute myelogenous leukemia new diagnosis, weakness, acute kidney injury	2	3	II, IS	Yes
30	63 F	White & AI/AK	Carpal spasms, hypocalcemia, hypomagnesemia, chronic severe PCM, adult FTT	0	2	II, IS	No
31	72 M	White	Encephalopathy, weakness, nausea and vomiting, pneumonia, adult FTT	1	4	II, IS, IL	No
32	64 F	White	Thiamine deficiency post bariatric surgery requiring IV thiamine infusion	0	3	II, IS	No
33	73 M	White	Pneumonia with sepsis, new onset CHF with reduced ejection fraction, hyponatremia, lower extremity wounds, atrial flutter with rapid ventricular response, acute thrombocytopenia, severe PCM	1	5	II, IS	No
34	76 M	White	Cellulitis with septic shock, chronic leg wounds, encephalopathy, severe PCM	1	5	II, IS, IL	Yes
35	75 M	White	Encephalopathy, hallucinations, weakness & falling, severe PCM, acute lymphoma, UTI, starvation ketoacidosis, dehydration, adult FTT	2	3	II, IS	Yes

36	73 M	White	CHF exacerbation, aortic stenosis, acute hypoxic respiratory failure, weakness & falling, decubitus ulcers, adult FTT, encephalopathy	1	3	II, IS	No

Abbreviations: #=Number of; AA = African American; AFIB= atrial fibrillation; AI/AK = American Indian or Alaska Native; AIC=acute Inflammatory condition; AKI=acute kidney injury; AVM=arteriovenous malformation; CHF=congestive heart failure; CIC=chronic Inflammatory condition; CLL=chronic lymphocytic leukemia; EF = ejection fraction; F= female; FTT = failure to thrive; G = gender assigned at birth; HIT=heparin induced thrombocytopenia; M =male; NH/PI = native Hawaiian or other Pacific Islander; N/V=nausea and vomiting; NSTEMI=non ST-elevation myocardial infarction; PCM=protein calorie malnutrition; RF=respiratory failure; RVR = rapid ventricular response; UA=unavailable; UTI = urinary tract infection; VT=ventricular tachycardia . 2. Probable etiologies of TD: II = insufficient intake; IS = inflammatory stress; IL = Increased losses

Table S2. BMI, weight change, biomarkers of malnutrition and inflammation, magnesium deficiency, and associated signs of malnutrition.

Case	BMI (kg/m2)	Non-volitional weight loss (%)	Albumin g/dL [3.5-4.7]	Prealbumin mg/dL [18-38]	Reported energy intake	Functional status	PE findings of M or SC fat loss
1	22.8	6.8% in 6 weeks	2.6	NA	Equivocal	Reduced	Yes
2	18	7.3% in 5 months	2.2	14	Insufficient	Reduced	Yes
3	27.8	14% in 5 months	4.6	NA	Insufficient	Reduced	Yes
4	22.6	6.3% in 6 months	3.3	NA	Insufficient	Reduced	Yes
5	22.7	no change	2.8	NA	UA	Normal	Yes
6	21.7	21% in 4 months	3.2	7	Insufficient	Reduced	Yes
7	30.3	no change	2.4	7	Insufficient	Reduced	Yes
8	31.5	7.1% in 4 months	2.3	10	UA	Reduced	No
9	24.4	10.5% in 6 months	2.9	NA	Insufficient	Reduced	UA
10	21	13% in 7 months	2.6	NA	Insufficient	Reduced	Yes
11	17.5	7.8% in 7 months	3.4	NA	Insufficient	Reduced	UA
12	21.5	11.8% in 2 months	2.7	NA	Insufficient	Reduced	UA
13	16.2	17.8% in 3 months	4.0	12	Insufficient	Reduced	Yes
14	27.4	no change	4.2	NA	UA	No change	Yes
15	20.3	UA	2.6	NA	Insufficient	Reduced	UA
16	27.6	no change	3.6	NA	Insufficient	Reduced	UA
17	34	UA	3.1	NA	Insufficient	Reduced	UA
18	29.9	6.4% in 6 months	2.1	NA	Insufficient	UA	UA
19	20.5	8.9% in 6 months	3.6	NA	Insufficient	UA	Yes
20	32.9	6.3% in 6 weeks	4.2	NA	Insufficient	UA	UA
21	24.3	12.3% in 4 months	3.0	NA	Insufficient	Reduced	Yes
22	30.8	Gained 4%	3.3	NA	No change	Reduced	UA
23	18.6	7.4% in 6 months	2.4	8	Insufficient	Reduced	Yes
24	23.2	15.7% in 9 weeks	2.2	NA	Insufficient	Reduced	UA
25	26.6	19.5% in 6 months	1.9	NA	Insufficient	Reduced	Yes
26	26.3	10.2% in 4 months	3.0	NA	UA	Normal	UA
27	43.7	7.3% in 4 months	3.7	NA	UA	Normal	UA
28	24.8	13% in 3 months	2.7	NA	No change	Reduced	Yes
29	24.9	14.1% in 8 months	3.9	NA	UA	Normal	UA
30	15.8	7.9% in 6 months	3.0	12	Insufficient	Reduced	Yes
31	33.3	3.2% in 6 months	3.9	NA	UA	Reduced	UA
32	33.7	22.7% in 4 months	3.9	NA	Insufficient	Normal	UA
33	29.9	No change	2.6	6	Insufficient	Reduced	Yes
34	28.3	17% in 2 months	2.3	7	UA	Reduced	UA
35	16	34% in 7 months	2.8	6	Insufficient	Reduced	Yes

36	40.6	13.7% in 10 months	3.1	16	Insufficient	Reduced	UA

BMI – body mass index; M – muscle; PE – physical exam; SC – subcutaneous; UA – unavailable

Red color = abnormally low

Table S3. Signs and Symptoms of Thiamine Deficiency.

Case	Weakness or falling	Neuro-psychiatric	Gastro-intestinal symptom	Ataxia	Poly-neuritis	Edema	Ophthal-moplegia	Non-ischemic CHF
1	+	+	+	+		+		
2	+		+			+		+
3	+	+	+		+		+	
4	+	+	+					
5								
6	+	+		+				
7	+	+	+	+	+	+		
8	+	+	+	+			+	
9	+	+						
10	+		+					
11	+					+		+
12	+	+	+					
13	+		+	+				
14		+						
15	+	+						
16	+	+	+	+				
17	+	+	+	+				
18		+	+	+				
19		+						
20		+						
21	+	+		+				
22	+			+				
23	+	+	+	+				
24	+	+	+					
25	+	+		+				
26								
27								
28	+	+						
29			+					
30	+	+						
31	+	+	+					
32		+			+		+	
33	+		+	+		+		+
34	+	+	+	+	+			
35	+	+	+		+		+	
36	+	+		+				
Totals/%	27(75%)	26 (72%)	19(53%)	15(42%)	5(14%)	5(14%)	4 (11%)	3(8%)

Neuropsychiatric symptoms include encephalopathy or delirium, delusions, hallucinations, agitation, odd behaviors, personality change, or seizures. GI symptoms include otherwise unexplained anorexia, nausea, gastroparesis, constipation, small or large bowel ileus. Ataxia was described as unsteadiness or ataxia by physician, or physical and occupational therapists. Edema was only included if no prior history of right or left heart failure, renal or liver failure. CHF is congestive heart failure.
